# Health vulnerabilities of undocumented central and eastern European migrants in Switzerland

**DOI:** 10.1016/j.jmh.2025.100327

**Published:** 2025-03-25

**Authors:** Zsolt Temesvary, Sabrina Roduit, Matthias Drilling

**Affiliations:** University of Applied Sciences and Arts Northwestern Switzerland, 4132 Muttenz, Hofackerstrasse 30, Switzerland

**Keywords:** Migration, Destitution, Central and eastern europe, Vulnerability, Homelessness

## Abstract

Destitute Central and Eastern European migrants, including homeless people, beggars, and sex workers, are in a highly vulnerable position in Switzerland. In the absence of residence permits, their access to health services and insurance is severely limited, and they suffer from institutional discrimination in Swiss medical facilities. The aim of this study is to examine the forms of health vulnerabilities of destitute mobile Eastern European citizens in Geneva and Zürich. To do this, we carried out narrative-biographical interviews with destitute migrants (*n* = 38) on their level of access to medical facilities and insurance. The results confirm that our respondents face severe vulnerabilities in accessing medical services and insurance mechanisms in Switzerland. This tendency is exemplified in the paper through the respondents' experiences of psychiatric disorders and substance abuse. Destitute migrants often transfer health vulnerabilities from their home countries. They mostly receive therapies and medicines in their countries of origin but remain untreated in Switzerland after arrival. Without Swiss health insurance, they turn to medical services only in cases of emergency, and even then, they are either rejected or discharged after very brief treatments. This dangerous combination of individual and systemic health vulnerabilities greatly exacerbates the disadvantages of destitute Eastern Europeans and hampers their integration into Swiss society.

## 1 Introduction

The neoliberal health systems of wealthy Western countries such as Switzerland and the United States often fail to alleviate the health disadvantages of highly vulnerable people (Burton-Jeangros and Fargnoli, 2023), such that entire social groups with serious medical needs can remain without proper care (Cullati et al., 2018). In Switzerland, many undocumented migrants were excluded from social and medical support during the first wave of the COVID-19 pandemic (Götzö et al., 2021). The Swiss system was not ready to vaccinate and treat undocumented people, mostly for administrative reasons. Even when free vaccines were available to all, undocumented migrants often did not know how to obtain them without proper information. In addition, they regularly avoided taking advantage of free medical services, as they were afraid to be registered and deported after revealing their undocumented status (Christen and Kurt, 2021; Burton-Jeangros and Fargnoli, 2023).

The Swiss system of healthcare is based on cooperation between private insurers and private or semi-private medical services such as hospitals, clinics, and general practitioners. Central government and cantons merely preside over the regulation of medical services and the occasional support of people who are unable to pay the market price for private insurance programmes. Swiss residents are obligated to have compulsory medical insurance, which they can supplement with various elective options (such as dental care or higher-level services such as a private room in hospitals). The price of basic insurance is a flat rate, which means that different income groups must pay the same price regardless of financial status. The price of insurance varies based on the given insurance company, the canton of residency, and the franchise (self-participation) of insured people. Only the poorest people, such as refugees or people receiving a social allowance or minimum pension, are eligible for state compensation to cover insurance fees.

Destitute undocumented Central and Eastern European (CEE) migrants, who include beggars, street sex workers, and street musicians, belong to the most vulnerable social groups in Switzerland. Approximately 30,000 undocumented CEE migrants currently live in Switzerland, and their number is steadily growing (Epple and Schär, 2015). According to the first Swiss homeless count, nearly a quarter of all homeless people living in Swiss cities originate from Central and Eastern Europe (Dittmann et al., 2022), and the proportion of CEE migrants among sex workers and beggars is between 70 % and 80 % (Rathgeb, 2013; Temesvary, 2022). Estimating the exact number of destitute CEE migrants is very difficult, as they are often not counted in the official migration statistics, since most of them live in Switzerland without a residence permit and are thus invisible to Swiss authorities. As EU citizens, destitute CEE migrants are generally permitted to stay in Switzerland for 90 days, after which they become undocumented if they cannot present work and rental contracts at Swiss immigration offices to legalise their status (Christen and Kurt, 2021).

Destitute CEE migrants often live in mass accommodation, in self-made temporary camps, or on the street, suffering from hunger and cold (Colombo et al., 2016; Temesvary, 2019). In the absence of residence permits, they are not eligible for Swiss health and social services (apart from a few emergency solutions) and are not supported by the system of social assistance (Christen and Kurt, 2021; Temesvary, 2022; Colombo et al., 2016). Destitute undocumented migrants are also not eligible for forms of support and services dedicated to refugees and asylum seekers, as they are treated as EU tourists whose medical insurance must be covered in their home countries.

According to previous studies, the reasons for the migration of destitute CEE citizens are mostly economic. In Switzerland, temporary jobs or atypical activities (such as begging or playing street music) can provide enough money in a few months for destitute CEE migrants to sustain themselves and their families for the rest of the year, which they spend in their home countries (Colombo et al., 2016; Scholten and van Ostaijen, 2018). As their income is very low (at least by Swiss standards), invisible, or both, destitute CEE migrants are generally not subject to taxation in Switzerland. However, like all other temporary or permanent Swiss residents, they are obligated to pay mandatory health insurance in order to receive health services. According to various data collection projects in Basel, Geneva, and Zürich (Temesvary, 2022; Colombo et al., 2016; Temesvary et al., 2021), destitute undocumented migrants are unable to pay expensive Swiss insurance fees.

Additionally, as most destitute migrants spend months or even years in Switzerland, their social insurance in their countries of origin expires, making them ineligible for health services in their home countries too (Temesvary, 2022; Roduit, 2020). As a result, highly vulnerable and impoverished migrants remain uninsured in both their home countries and Switzerland. In the absence of Swiss medical services, they sometimes use the health services of neighbouring countries such as Germany or France, as previous studies by Temesvary (Temesvary, 2022) in Basel and by Roduit (Roduit, 2020) in Geneva have described. These neighbouring countries sustain social security–based health systems and provide more accessible services for impoverished “EU tourists” than Switzerland does. Furthermore, destitute CEE migrants can travel home to receive treatment in some non-urgent cases if they still have health insurance in their countries of origin.

Considering the above-mentioned conditions, our paper seeks to answer the following question: *Which health vulnerabilities do destitute CEE citizens face in Switzerland, and how do Swiss health services respond to these vulnerabilities?* To answer this question, we follow a constructivist research paradigm with an open-ended inquiry. First, we examine systemic vulnerabilities (Bonvin et al., 2023) such as the availability and accessibility of Swiss health services and health insurance schemes. We subsequently explore experiences with the usage of medical systems in the home countries. Finally, we focus on individual health vulnerabilities using the examples of psychiatric diseases and substance abuse.

## 2 Theory and context

The comprehensive concept of resources (reserves) and vulnerabilities focuses on the dynamics and status of the well-being of individuals and communities (Spini and Widmer, 2023). Psychological, health, and social resources consist of material and nonmaterial goods, skills, conditions, networks, and privileges that contribute to a steady state which provides protection from stressors and diseases that endanger well-being (Thompson, 2021). Health reserves and vulnerabilities are affected by genetic and biological conditions, family and work circumstances, and social factors such as the living environment (Burton-Jeangros et al., 2015). These internal and external determinants intersect and can reinforce reserves or exacerbate vulnerabilities, based on the cumulative advantage and disadvantage model of Dannefer (Dannefer, 2003). Social inequalities (for example in housing and working conditions) contribute strongly to the exacerbation of health vulnerabilities. Health policies and institutions should diminish these vulnerabilities and secure the access of vulnerable groups to medical services (Cullati et al., 2018).

Health vulnerabilities are not always related to objective systemic conditions (such as the availability of services) and other objective indicators. The subjective perception of health vulnerabilities is at least as important as the objective dimensions in understanding the sophisticated intersection of health (and other) vulnerabilities. The subjective perception of health is highly affected by cultural dimensions that are deeply rooted in communities (Cullati et al., 2018). Besides the impact of family and community, the individual perceptions of health regarding a person's medical history, experiences, attitudes, and other factors (e.g., how they tolerate pain or the lack of hygiene) are also important elements of subjective health vulnerabilities (Burton-Jeangros and Fargnoli, 2023; Roduit, 2020).

This paper contemplates health vulnerabilities through access to health services and insurance schemes. Access to medical services has multiple approaches in the international literature (Aday and Andersen, 1974), such as financial, geographical and political dimensions. This paper examines access to medical services from both the systemic and individual contexts, emphasizing the systemic framework while exploring the individual dimensions of access through the experiences of patients with psychiatric disorders and substance abuse.

## 3 Methods and data collection

### 3.1 Sampling

Participants for the expert interviews were recruited from homelessness and medical services, where the client interviews were also conducted. During recruitment, we approached experts in various positions, ranging from case workers to managers.

The client interviewees were recruited via a snowball method. We explicitly required respondents to answer our questions and then introduce us to other potential participants for further interviews. The support of social workers was very important in our selection of destitute CEE migrants. The combination of the snowball method and the approach of reaching interviewees through social workers contributed to the randomness of the target group. For the client interviews, we selected people arriving from CEE countries that were EU member states because of the varying eligibilities of EU and non-EU citizens in Switzerland. Our selection criteria were as follows: (1) CEE citizenship, (2) a past or current undocumented status in Switzerland, (3) financial poverty and (4) experience with homelessness.

Financial poverty was identified based on the Swiss subsistence minimum defined by the Federal Office of Statistics, which was 2259 CHF per person per month in 2021. We considered people destitute if they lived in financial poverty and could not sustain themselves without the support of other people or organisations (Harris-White, 2005). Homelessness was defined based on the ETHOS (European Typology of Homelessness and Housing Exclusion) typology developed by FEANTSA, an international homelessness organization. This categorization considers people homeless if they are roofless, lack housing, or their accommodation is insecure or inadequate (Amore et al., 2021).

### 3.2 Research methods, data collection and processing

During data collection, we applied qualitative methodology in the form of explorative expert interviews and qualitative narrative-biographical interviews with destitute CEE migrants. The data collection started in April 2021 and was completed in November 2022.

In the first wave of data collection, we carried out 16 expert interviews with frontline workers for social and medical service providers (such as social workers, counsellors, mental health experts and managers) working directly with destitute CEE migrants. These expert interviews helped us to understand the systemic context of health vulnerabilities from the viewpoint of services. Expert interviews were conducted prior to the client interviews, and the information gathered from the expert interviews was used to inform the preparation of the client interviews. The expert interviews were conducted using an interview guide that focused on the following categories: (1) general perceptions of the health conditions of destitute migrants, (2) availability and accessibility of medical services, (3) primary challenges concerning availability and developmental potential, and (4) the effects of the COVID-19 pandemic on destitute CEE migrants. All expert interviews were recorded online or in separate interview rooms. The interviews were then processed using Atlas.ti (a computer-based data processing software), following the qualitative content analysis method outlined by Mayring (Mayring, 2000). Expert interviews are presented in the paper in an interpretative manner, organized under the main chapters based on the categorization of the client interviews.

In the second wave, researchers carried out 38 narrative-biographical interviews with destitute undocumented CEE migrants in Zürich and Geneva, the two largest cities in Switzerland. During the narrative interviews, respondents were free to discuss their life conditions, and interviewers only marginally directed them towards the main research topic of individual and subjective health vulnerabilities (Götzö et al., 2021; Colombo et al., 2016; De Coulon et al., 2015).

Most client interviews were conducted at social institutions, such as soup kitchens, day-care services and counseling stations. Some interviews took place in public spaces, while a few were held in ambulatory medical services. The interviews were conducted and processed by research fellows at the FHNW and by external colleagues with special language skills. We conducted the interviews in German, English, French, Romanian and Hungarian. All interviews were recorded for transcription in separate rooms. Recordings were permitted by the respondents, and interviews were not conducted when digital recording was not allowed.

Similar to the expert interviews, client interview transcripts were analyzed using Atlas.ti. For the analysis, we applied Mayring`s method of qualitative content analysis, using main categories such as the prevalence of chronic diseases and injuries, access to Swiss healthcare services and health insurance schemes, traveling home for care, and experiences with psychiatric disorders and substance abuse. Codes were then organized under these previously created categories. Researchers in Geneva and Zürich worked separately on a few interviews, then shared project bundles generated from Atlas.ti and merged, selected, and refined the codes into a unified coding scheme that was applied throughout the analysis of the remaining interviews (Table 1).

**Table 1:** Methods applied and main characteristics of the sample.

	Client interviews	Expert interviews
Sample size	38 participants	16 participants
Recruitment method	Narrative-biographical interviews	Explorative expert interviews
Inclusion criteria	Undocumented CEE migrants living in homelessness in Zürich and Geneva	Frontline workers at homelessness services in Zürich and Geneva
Age range	19–63 years	34–57 years
Gender	53 % male, 47 % female	44 % male, 56 % female

Thematic saturation was achieved as the analysis of the interviews progressed, with recurring themes such as access to services, insurance schemes, traveling home, and experiences with psychiatric disorders. After coding and analyzing approximately 20–25 interviews, we determined that the sample size was sufficient, as all key categories were captured, ensuring comprehensive coverage of the research objectives (Table 2).

**Table 2:** Main characteristics of the respondents in the client interviews (*n* = 38).

Category	N	%	Category	n	%
Age			Citizenship		
>31	10	26	Romania	22	58
31–45	16	42	Hungary	7	18
46–50	11	29	Bulgaria	3	8
<61	1	3	Other (SK/CR/PL)	6	16
Gender			Residence permit		
Male	20	53	Without permit	31	82
Female	18	47	With permit	7	18
Family status			Ethnicity		
Single	22	58	Roma	24	63
Partnership	16	42	Non-Roma	14	37

The qualitative research data in this paper is presented in an interpretative manner (Wiesner, 2022). Apart from a few summarizing tables of the main results, the data is analyzed interpretatively, focusing on the research question, the main categories used in the sequential analysis, and the paper's theoretical framework (Table 3).

**Table 3:** Health vulnerabilities of destitute CEE migrants (*n* = 38).

Category	n	%	Category	n	%
Perceived good health			Having chronic disease		
Yes	29	76	Yes	7	18
No	9	24	No	31	82
Insurance in Switzerland			Hospitalised in Switzerland		
Yes	8	21	Yes	13	34
No	30	79	No	25	66
Receiving care in CEE			Insured in CEE		
Yes	11	29	Yes	20	53
No	27	71	No	18	47
Diagnosed with psychiatric disorders			Experiencing substance abuse		
Yes	8	21	Yes	11	29
No	30	79	No	27	71

The research design, including the interview guides and data collection methods, was developed based on the ethical guidelines of Swissethics, an ethical board that forms ethical guidelines for Swiss universities. The research plan and data collection methods adhered to the standards of Avenir Social (Swiss Association of Social Workers), the Ethical Board of the University of Applied Sciences Northwestern Switzerland, and the Ethical Committee of the supporting research centre, NCCR LIVES. For the publication of our qualitative results, we applied the SRQR (Standards for Reporting Qualitative Research) guidelines and subsequently used the checklist provided by this guide (Brien et al., 2014).

## 4 Results

### 4.1 Main characteristics of the respondents

Female and male respondents were evenly represented in the interviews, with 47 % (*n* = 18) female and 53 % (*n* = 20) male participants. The target group was relatively young. One-fourth (26 %, *n* = 10) of respondents were under 31 years old, while one-third (24 %, *n* = 16) were between 32 and 45 years old. In terms of nationality, more than half (58 %, *n* = 22) of respondents were from Romania, followed by Hungary (18 %, *n* = 7) and Bulgaria (8 %, *n* = 3). The majority (63 %, *n* = 24) of interviewees belonged to the Roma ethnic minority. The Roma people were overrepresented among the participants, with their proportion among destitute CEE migrants being much higher than their share of the population in their countries of origin (which ranges from 8 % to 12 %) (Bernát, 2016). Regarding housing and residency conditions, 76 % (*n* = 29) of respondents were homeless at the time of data collection in Switzerland, according to ETHOS typologies, and 82 % (*n* = 31) of the interviewees were residing without a residence permit in Switzerland.

### 4.2 Access to Swiss health insurance

Without residence permits, it is almost impossible to apply for health insurance, and consequently, only 8 of the 38 interviewees (21 %) in Switzerland had health insurance. Additionally, flat-rate insurance fees disproportionally burden poor people, as they must turn a large share of their income towards insurance fees. Moreover, the most vulnerable people avoid care for financial reasons, because of the high out-of-pocket expenses incurred by the patient (Roduit, 2020). In rare cases, destitute migrants intentionally choose illegal employment to avoid taxation and high health insurance burdens. For instance, a former sex worker, working now as a part-time cleaner, gave up her temporary residence permit and continued working illegally, as she could not pay taxes and contributions out of her low income.

*If you have papers without a job, you still need to pay taxes and health insurance. For what? I do not have 300 francs for a health insurance that I do not even use. It is a luxury that I cannot afford. (woman, Hungary, 51)*

If destitute CEE migrants lack Swiss health insurance, they are forced to rely on the support of NGOs that can pay their hospital bills. There are no permanent state or cantonal programmes to cover these debts; however, there are some initiatives that provide support for uninsured people.

*I was stabbed three times in the belly at the railway station and spent nearly two months in the hospital afterward. An NGO called Victim Support covered my medical bills (man, Poland, 19).*

Social workers in frontline services mentioned that some CEE citizens forgot to cancel their Swiss health insurance when they returned to their home countries, and the debts from the unpaid insurance fees accumulated in Switzerland. As a result, when they returned to Switzerland, they faced serious debts, the payment of which caused a lot of difficulties for social organisations. People without insurance can be viewed by medical facilities as being risky and expensive patients, and therefore hospitals often only provide them with emergency services and discharge them as soon as the urgent treatment ends (Blaser et al., 2021). Receiving rehabilitation is practically unattainable for destitute CEE migrants without health insurance, and vulnerable people are discharged very early without follow-up examinations.

*Even if they are in psychosis and are hospitalised, they are discharged in 24* h *without medical insurance. This affects not only Eastern Europeans but other uninsured EU migrants, too. (social worker at a daycare service)*

### 4.3 Access to health services in Switzerland

The qualitative interviews showed that only 5 of the 38 respondents (13 %) used general medical services (such as general practitioners or ambulatory services) in Switzerland, and one-third (13 people) of them mentioned that they needed a general medical service but could not receive it for several reasons (mostly because of the missing health insurance). Our data showed that 8 respondents (21 %) attended hospitals in Switzerland, and only 5 person (13 %) used general services. This confirmed our suspicion that destitute CEE migrants in Switzerland primarily accessed medical services only in cases of urgent needs, particularly for severe pain or injuries. These cases often include serious injuries where treatment is necessary and cannot be postponed. Injuries regularly happen at work, as destitute migrants are often employed illegally under dangerous conditions as construction workers, kitchen aids, or harvest workers.

*In 2018, I suffered a serious hand injury. I was working on a scaffold when I fell, right on my hand. It was broken twice […]. I was taken to the hospital by my colleagues, where I received first aid. (man, Romania, 33)*

Most destitute CEE citizens faced a serious shortage of medical care and treatment in Zürich and Geneva. Free health services are generally not available for poor undocumented people in Switzerland, apart from a few ad hoc forms of support organised by local NGOs. Services such as CAMSCO in Geneva and Ambulatorium in Zürich offer treatment for destitute undocumented migrants for free or at a reduced price. These services are limited to counselling and short-term therapies. The costs of such treatments are mostly paid by social organisations or public hospital funds.

*If you have insurance, you don't need help. If you don't have insurance, you must go to CAMSCO, and they give you vouchers and you can have your teeth removed. (woman, Romania, 31)*

Swiss hospitals reject destitute undocumented CEE migrants unless the treatment is for emergency or epidemic reasons. Illnesses not requiring urgent care are usually not treated by Swiss medical facilities.

*Once I tried to go to a clinic that was free for all, but I was sent away, and they told me to go back to my Slovakian doctors, as they treat only injuries and illnesses that require immediate intervention. (man, Slovakia, 40)*

Hospitals and clinics treating uninsured people regularly send the medical bills directly to the people or social organisations with whom they were staying before their hospitalisation, such as homeless shelters or day-care services. Social organisations attempt to help their clients through private donations and cantonal support, but their financial capabilities are very limited. Accumulating medical bills often push destitute people into a debt spiral as they must pay unsettled debts immediately after finding a job. A social counselling station in Zürich has the ability to effectively support its clients with a combination of private donations and state support.

*A lot of destitute Central and Eastern Europeans are sent to us by hospitals. They receive care but cannot pay the bills without insurance. So they are sent to us by hospitals or by the insurers, where they have serious debts because of unpaid bills. (manager at a counselling station for sans-papiers)*

Understanding the Swiss healthcare system, with its multiple insurers and numerous services, is extremely complicated for newcomers (Roduit, 2020). Even if they are allowed health insurance, destitute CEE migrants often do not understand the Swiss system of private services and insurance, which is markedly different from the social security systems in CEE countries. In the absence of adequate language skills, and in light of the difficulty of adapting to their new sociocultural environment in Switzerland, many destitute CEE migrants do not trust the Swiss healthcare system. They are also often afraid of the costs they must pay after leaving hospitals.

*There is mistrust among destitute Eastern European migrants towards the Swiss medical services. A Roma woman, for example, left the emergency station a day after she had been admitted despite her serious heart problems. (social worker at a daycare service)*

According to our expert interviews carried out with service managers at the City Social Departments of Zürich and Geneva, medical care for destitute CEE “tourists” is not the responsibility of the city, and the Social Department cannot take over the expensive costs of therapies for every uninsured foreigner. The administrative managers stated that if destitute undocumented people do not need urgent help, they must return to their home countries where they are insured. As the number of unpaid medical treatments is very high, political debates about the responsibility of the cantons have become very intense in the past few years. The situation was further exacerbated during the recent COVID-19 pandemic, during which a lot of uninsured CEE migrants were treated in Switzerland.

*Medical care is not the responsibility of the city – if they need constant help, they must return to their home countries. (manager of the city's social services, Zürich)*

The COVID-19 pandemic led to new and more precarious living conditions for destitute CEE migrants in both Switzerland and their countries of origin. Their living circumstances became even harder in their home countries, motivating them to travel abroad to find better opportunities. However, job possibilities became very limited in Switzerland, too. Sex work, for instance, was prohibited in most Swiss cantons for reasons of hygiene, while begging and street music were also no longer viable as people disappeared from public places during the lockdown. Positions traditionally occupied by women in gastronomy and home care were also limited as a result of the pandemic (Götzö et al., 2021).

*COVID functioned as a booster in the migration of destitute CEE people as life became more unbearable in their home countries. (social worker at a daycare service)*

Fortunately, local charity programmes and private donations from the Swiss Solidarity Fund can support some field institutions in diminishing the social exclusion of highly vulnerable groups. Frontline social work organisations, such as soup kitchens, day-care services, night shelters, and counselling stations, are mostly sustained by NGOs, and their survival depends on private and public donations. These NGOs are often poorly financed, and they work with many volunteers, as they cannot admit qualified social workers.

*A lot of sans-papiers have lost their jobs during COVID. They need urgent social assistance. We received a lot of support from private persons,* e.g.*, in the form of food tickets, or they took over the health insurance fees. (manager at a counselling station for sans-papiers)*

Even if destitute CEE patients receive care in Switzerland, it is clear from the biographical interviews that most patients were discharged after a short course of medical treatment, they did not receive any rehabilitation, and they were not cared for after leaving the hospital.

*I was taken to the hospital. I spent three weeks there. I said that I was hearing voices and had anxiety. After three weeks I was simply discharged onto the street. (man, Hungary, 41)*

Very early discharges are not medically underscored, and they often lead to a “swing door effect”, as people soon return to medical facilities because their diseases are not cured; rather, their symptoms are temporarily alleviated (Blaser et al., 2021). This practice imposes further costs on medical facilities that would be avoidable with adequate treatment.

### 4.4 Receiving healthcare in home countries

Due to the absence of available healthcare services in Switzerland, 11 respondents (29 %) travelled to the home countries for medical care. However, some respondents, who wanted a treatment in the countries of origin, no longer had insurance in Eastern European countries, or they could not afford the high costs of travel. The most such journeys that did occur were for dental and gynaecological treatments, which could be planned for.

*So, I finally returned to Romania for the care. It took two weeks as I had to go back more times. The health system in Romania is not the best [laughing], but they did a good job with my teeth. (woman, Romania, 36)*

Besides easier accessibility, the other reasons for respondents using services in their home countries were language (they understood the doctors) and general trust in CEE medical personnel (and lack of trust in Swiss medical services). For example, a Slovakian middle-aged man was afraid that his data would be forwarded to the Swiss immigration authorities, and he would be deported after receiving care.

*Apart from that, I am healthy and do not want to go to the hospital as I do not trust Swiss doctors. They forward your data to the police, and you will be deported with a short notice. (man, Slovakia, 45)*

### 4.5 Experience with psychiatric disorders

Serious psychiatric disorders such as depression, neurosis, schizophrenia, and anxiety occur relatively frequently among destitute CEE migrants. Eight of the 38 respondents (21 %) had a diagnosed psychiatric illness, and the number of undiagnosed cases can be even higher. Street sex workers and the homeless are particularly at risk due to their extreme living and working conditions (Dittmann et al., 2022; Blaser et al., 2021; Gregoris et al., 2020). Some of our interviewees were originally diagnosed and treated in their home countries but went untreated and stopped taking medicines after arriving in Switzerland. Psychiatric disorders deepen the vulnerability and exacerbate the social marginalisation of destitute undocumented people (Balasuriya et al., 2020). Homelessness and sex work often result from serious psychiatric disorders, and people land on the street because of the social implications of such disorders (Lutz et al., 2021).

*They have often serious psychiatric disorders. Those women who work while suffering from psychosis are not professional sex workers; they simply have no other choice to sustain themselves. Mentally ill sex workers often need to leave brothels, and thus the street is the only place where they can work. (social worker at a counselling station for sex workers)*

Although one-fifth (8 people) of our interviewees had previously been diagnosed with psychiatric illnesses (mostly in their countries of origin), only half of them (4 people) had received psychiatric care in Switzerland. Despite their limited access to Swiss psychiatric services, most respondents did not consider their lack of psychiatric care as a problem, and they expressed the belief that psychiatric disorders were not “real” diseases.

*- Do you have any illnesses?*

*- No, I am healthy. But I hear voices in my head. I have been under psychiatric treatment for 7 years and I also receive medicines. (man, Hungary, 41)*

Most interviewees who previously received psychiatric treatment in their home countries had been diagnosed with schizophrenia and/or chronic depression. They had usually received proper psychiatric care in their countries of origin but could not continue their treatment in Switzerland.

*I suffered so much that I fell into depression. At first, it was just a bad mood, but later, suicidal thoughts came too. I spent a few weeks in hospital because of burn-out and other problems. (man, Hungary, 24)*

Half of the respondents diagnosed with psychiatric disorders in their home countries did not receive adequate care in Switzerland, while the other half only received emergency care in cases of psychosis. Going without psychotherapy and medication contributed to the exacerbation of their psychiatric illnesses and increased their risk of psychosis.

*I have no insurance, so I do not have medicines now. I do not know how to get my pills here, but they will hopefully help me with it. Depression is a terrible thing; if I cannot have my medicines, I collapse mentally like an old house. (man, Romania, 27)*

The seriousness of a lack of medication is shown by the fact that a few respondents had to be transported to psychiatric clinics for forced treatment when they experienced psychosis and endangered other people in public spaces. A Hungarian man with schizophrenia, for instance, spent weeks in custody before he was transported to a psychiatric clinic, because he attacked a passenger in a tram while in a state of psychosis. In the absence of adequate mental health services, some of the interviewees tried to sustain their consultations with their Eastern European doctors or solicited family members to send them medicines by post. A man suffering from schizophrenia originally organised his medication through his mother, who regularly posted pills to him from Romania, but this was not viable in the long term.

*In the beginning, when as I was in Chur, my mum sent me my medicines by post. She got my doctor to prescribe the pills and then she sent them to me every single month. Since I have been in Zürich, I have had no medication. (man, Romania, 41)*

The lack of proper medication and treatments affects not only people with psychiatric illnesses but also those suffering from other chronic diseases. For example, one HIV patient obtained some drugs from an ambulatory HIV/AIDS clinic in Zürich but not enough of them to secure his long-term treatment. This insecurity regularly leads to anxiety for many patients living without Swiss health insurance.

*I went to the hospital after coughing for two weeks. I am HIV positive. […] My doctors said that I cannot stop taking my medicines or I will die. I have only a few medicines left, and I am in a panic. (man, Romania, 31)*

### 4.6 Experience with substance abuse

Eleven (29 %) interviewees talked about having problems with substance abuse that considerably affected their daily life. Additionally, most interviewees suffering from addiction combined alcohol with various illicit drugs. The influence of a partner or peer group was very important in the development of substance abuse.

*My girlfriend had a lot of problems, she used drugs. Okay, we both used drugs, we did stupid things together. After that, we broke up; I stopped drugs but continued drinking. (man, Hungary, 24)*

Among destitute CEE migrants, addictions usually start in their home countries at a very young age, with cheap drugs such as “herbal” (a synthetic drug often used in Eastern Europe), painkillers, and other materials. Then, when in Switzerland, migrants occasionally switch to “Western” drugs (such as heroin or cocaine), further exacerbating their drug dependency.

*I used drugs at home, but only cannabis and other light drugs. In Zürich, I got involved with a very bad crowd and started using heroin every day. I want to distance myself from them, but I always end up back in this group. (man, Bulgaria, 29)*

Based on the biographical interviews, drug consumption is often rooted in unprocessed childhood trauma, including family violence, rape, and hunger. For instance, a young Romanian man who was raised in an orphanage, occasionally raped by the older boys, and forced to beg used drugs as a “medicine” to overcome childhood trauma. This kind of problem management only further exacerbates the precarious situation of dependent people.

*I started using drugs not to feel this shit life around me and not to think any more about my past. I went to the UK to leave this kind of life behind. But when I dream, I see pictures from my childhood, from my past. (man, Romania, 29)*

Psychiatric disorders and drug consumption often accompany each other. In the absence of medicines, destitute people with psychiatric issues often alleviate their symptoms by drinking and using illicit drugs. Several interviewees were arrested because of drug-related issues when they either sold drugs or committed crimes under the effect of illicit drugs.

*We often used drugs at that time. Once, I was involved in a robbery where a man was beaten and robbed. My friend ran away, but I was caught and sentenced to three years in prison. (man, Hungary, 35)*

Substance abuse not only worsens one's individual life prospects but also harms social relationships with family and friends. Besides poverty, loneliness and social isolation often trigger emigration from Central and Eastern Europe. However, addicts are unable to improve their living conditions and social relationships upon arrival in Switzerland, as substance abuse hampers their efforts to start a new life in a foreign country. Family and community networks are often eroded because of drug consumption and its accompanying problems, such as theft, deceit, and unpredictability.

*After I moved out of my mother's place, I went to live with my sister. I stole a few things when I needed money, and her husband threw me out after a week. Now, I feel ashamed of what I did. (man, Bulgaria, 25)*

Alcohol and illicit drugs often help to reduce the shame and tension destitute people feel when they beg or play music in public places. Because of this vicious circle of extreme poverty and excessive substance abuse, the more they beg, the more alcohol and drugs they use.

*I always drink when I play. I use alcohol as I feel ashamed for doing that. People watch me and think that I am a young man and I could work like the others. (man, Hungary, 24)*

## 5 Discussion

### 5.1 Main findings

Our paper was a first attempt to scrutinise the health vulnerabilities of destitute CEE migrants living in Switzerland. Nevertheless, our results underscore that the general health conditions of destitute CEE migrants are highly precarious. Due to their dangerous living and working conditions undocumented CEE migrants are highly exposed to psychiatric disorders, and substance abuse. Besides individual health vulnerabilities, they also suffer from systemic disadvantages that are visible through their severely limited access to medical services and insurance schemes. These dual health vulnerabilities, at both individual and systemic levels, impact a highly disadvantaged group who also suffer from other complex vulnerabilities in housing, employment, and social relationships.

High medical costs and the fear of being registered and deported keep most destitute CEE migrants away from Swiss medical facilities. They attend Swiss hospitals and doctors only if treatment is urgent and unavoidable. Psychiatric illnesses and extensive substance abuse are particularly relevant problems among destitute CEE migrants, and these disorders significantly hamper their chances of achieving successful social integration and labour market participation. Untreated psychiatric diseases, as well as drug and alcohol-related problems, are often exacerbated by precarious living and working conditions, and vice versa.

Apart from some injuries and emergency cases, most health problems that we identified in Switzerland had developed and been diagnosed in the interviewees’ home countries and then been “transferred” to Switzerland. Despite obvious health vulnerabilities, most participants characterised their subjective health status as good or satisfactory during the interviews. Most interviewees did not view psychiatric disorders and substance abuse as illnesses. This positive self-assessment was probably due to the relatively young age and good physical health of the interviewees.

### 5.2 Political implications

Swiss social policy is based on the welfare principles of subsidiarity, less eligibility, and locality (Wang and Aspalter, 2006). Like other studies (Colombo et al., 2016; De Coulon et al., 2015), our expert interviews underscore that decision-makers and administrative managers in cantonal social and health departments attempt to discourage “welfare tourism” by providing the least possible social rights and eligibilities for undocumented European migrants regarding their access to Swiss social and health services. As a result, thousands of undocumented CEE migrants remain without medical insurance and care in Swiss cities. As they often spend months or years in Switzerland as undocumented immigrants, they also often lose their Eastern European health insurance and cannot return to their home countries for care (Temesvary, 2022). There is currently no federal solution for providing medical care for undocumented EU migrants in Switzerland, but some cantons, such as Zürich, Lausanne, Basel, and Geneva, attempt to provide alternative forms of local support for destitute CEE migrants, allowing limited access to their local health and social systems.

Zürich, for instance, introduced a pilot program called the City Card to simplify access to emergency medical and social services during the pandemic, while Basel accepted a political decree about the support of destitute CEE migrants through basic services. These are promising local initiatives that can temporarily alleviate the vulnerabilities of undocumented migrants. However, it is very important for there to be a federal regulation on a minimum level of access to Swiss medical services for people without health insurance. Without political support for concrete social rights, destitute migrants remain powerless within the Swiss welfare system. The few NGOs supporting undocumented migrants suffer from financial problems, and their human and infrastructural conditions are deficient, as shown during the recent COVID-19 pandemic. Some NGOs organise temporary medical examinations and counselling for their clientele, but these services cannot satisfy the growing and complex needs of destitute migrants.

Our results highlight that destitute CEE citizens need special attention from the Swiss healthcare system. They have very poor access to healthcare in their home countries and Switzerland alike, and this deprivation leads to specific and cumulative health vulnerabilities. Our findings show the urgent need for more equity and solidarity in the Swiss healthcare system because destitute migrants are caught between two health systems (Switzerland and Eastern Europe), often resulting in them being rejected and left without medical care.

### 5.3 Where future research is needed

Even though the health vulnerabilities of migrants are mostly rooted in CEE countries, Swiss research projects examining the health conditions of destitute CEE migrants in Switzerland avoid analysing the development of health problems in their countries of origin. The evaluation of “original” health vulnerabilities at the micro-, meso‑, and macro-levels (such as environmental impacts, institutional racism against the Roma, and the malnutrition of children living in poverty) in the migrants’ home countries and their implications in Switzerland would be a huge asset in understanding the problem and supporting decision makers through the use of empirical data (Battaglini and Hasdeu, 2017). As part of such an evaluation, local CEE communities affected by migration should be examined by researchers to reveal the relationship between poor health and migration. For instance, tuberculosis (TB) occurs relatively often in destitute Eastern European communities such as Roma settlements and homeless camps, while this disease is almost forgotten in Switzerland. People suffering from TB become isolated, often lose their social relationships and jobs, and frequently become impoverished. We also found similar tendencies with people suffering from HIV/AIDS or psychiatric disorders. Health-based social exclusion can be a reason for migration for many people. Uncovering these comprehensive tendencies is a prerequisite for developing adequate services for people with health vulnerabilities in Switzerland.

### 5.4 Strengths and limitations of the study

During this study, we were able to involve a considerable number of experts and destitute migrants, providing a broad and reliable perspective on the key systemic and individual health vulnerabilities of the target group. Despite the pandemic, we gained access to major homelessness services and medical facilities, enabling us to collect a solid body of data. In doing so, we highlighted both the individual and systemic dimensions of health vulnerabilities, which can contribute to the development of better and more effective services and measures targeting destitute CEE migrants.

Despite its strengths, the current study has certain limitations. For instance, the data collected only covers the two largest Swiss cities and cannot be considered representative of the entire country. A limitation in data collection was that some individuals, particularly those avoiding authorities and homelessness services, could not be reached. This was often the case with destitute Roma living outside the selected cities, despite them being among the most vulnerable groups of poor migrants. Our interviews were carried out during and after the first wave of the COVID-19 pandemic, which created a unique and exceptional situation at the organizations. As a result, we could not observe how these organizations and their clientele functioned under "normal" conditions, rather than during a global pandemic.

### 5.5 Conclusion

Eastern European undocumented migrants face significant barriers in accessing Swiss medical services, with therapies restricted to emergencies such as accidents, injuries, or serious illnesses. Conditions like psychiatric disorders and substance abuse often remain untreated, leaving many without the care they need. Additionally, hospital stays are typically short, and patients are discharged without follow-up treatments, further exacerbating their vulnerabilities. As a result, many turn to the healthcare systems of their home countries as an alternative strategy to address their medical needs. These findings highlight the urgent need for more inclusive healthcare policies and support systems to address the unmet health needs of this marginalized group.

## CRediT authorship contribution statement

**Zsolt Temesvary:** Writing – review & editing, Writing – original draft, Visualization, Supervision, Resources, Project administration, Methodology, Investigation, Funding acquisition, Formal analysis, Data curation, Conceptualization. **Sabrina Roduit:** Writing – review & editing, Supervision, Resources, Project administration, Methodology, Investigation, Funding acquisition, Data curation, Conceptualization. **Matthias Drilling:** Writing – review & editing, Validation, Supervision, Methodology, Formal analysis, Conceptualization.

## Declaration of competing interest

The authors have no conflict of interests to declare.
